# Postglacial dispersal patterns and mitochondrial genetic structure of the Pyrenean desman (*Galemys pyrenaicus*) in the northwestern region of the Iberian Peninsula

**DOI:** 10.1002/ece3.3034

**Published:** 2017-05-15

**Authors:** Marina Querejeta, Angel Fernández‐González, Rafael Romero, Jose Castresana

**Affiliations:** ^1^ Institute of Evolutionary Biology (CSIC‐Universitat Pompeu Fabra)BarcelonaSpain; ^2^ Biosfera Consultoría Medioambiental S.L.OviedoSpain; ^3^ Calle Presidente Salvador Allende 13Santiago de CompostelaSpain

**Keywords:** dispersal, *Galemys pyrenaicus*, genetic diversity, Iberian Peninsula, isolation‐by‐distance, mitochondrial DNA

## Abstract

The genetic structure of small semiaquatic animals may be influenced by dispersal across both rivers and land. The relative importance of these two modes of dispersal may vary across different species and with ecological conditions and evolutionary periods. The Pyrenean desman (*Galemys pyrenaicus*) is an endemic mammal of the Iberian Peninsula with a strong phylogeographic structure and semiaquatic habits, thus making it an ideal model to study the effects of river and overland dispersal on its genetic structure. Thanks to different types of noninvasive samples, we obtained an extensive sampling of the Pyrenean desman from the northwestern region of the Iberian Peninsula and sequenced two mitochondrial DNA fragments. We then analyzed, using an isolation‐by‐distance approach, the correlation between phylogenetic distances and geographical distances measured along both river networks and land to infer the relative importance of river and overland dispersal. We found that the correlations in the whole area and in a large basin were consistent with an effect of overland dispersal, which may be due to the postglacial colonization of new territories using terrestrial corridors and, possibly, a more extensive fluvial network that may have been present during the Holocene. However, in a small basin, likely to be less influenced by the impact of ancient postglacial dispersal, the correlations suggested significant overall effects of both overland and river dispersal, as expected for a semiaquatic mammal. Therefore, different scales and geographical regions reflect different aspects of the evolutionary history and ecology of this semiaquatic species using this isolation‐by‐distance method. The results we obtained may have crucial implications for the conservation of the Pyrenean desman because they reinforce the importance of interbasin dispersal for this species in the studied area and the need to protect the whole riverine ecosystem, including rivers, upland streams and terrestrial corridors between basins.

## 1 INTRODUCTION

One of the greatest challenges in phylogeography is to understand how geography and ecology shape the genetic structure of species (Avise, 2009). Indeed, geography in particular can affect genetic structure in many ways, such as by inducing the fragmentation of populations and shaping the dispersal patterns among them (Manel, Schwartz, Luikart, & Taberlet, 2003). This is particularly important for riverine species, which usually have strict ecological requirements. When the river networks have suitable physical and ecological conditions for the species, they can favor connectivity of populations (Byrne, Quintana, Bolkovic, Cassini, & Túnez, 2015; Chaput‐Bardy, Lemaire, Picard, & Secondi, 2008; Paz‐Vinas, Loot, Stevens, & Blanchet, 2015). Rivers may also act as barriers to dispersal of aquatic species when their width, depth, and water regimes or the presence of water infrastructures are inappropriate for the species (Bartáková, Reichard, Blažek, Polačik, & Bryja, 2015; Byrne et al., 2015; Raeymaekers et al., 2008). Despite their interest, there are still few studies that have analyzed the effects of river systems on the genetic structure of semiaquatic mammals, in which their degree of dependency on the aquatic medium is crucial for understanding when rivers act as barriers or as dispersal paths (Furlan et al., 2013; Pagacz, 2016; Senn et al., 2014).

In this study, we focus on the Pyrenean desman or Iberian desman [*Galemys pyrenaicus* (Geoffroy, 1811)], a small semiaquatic mammal endemic to the Iberian Peninsula. The Pyrenean desman is highly dependent on specific features of the rivers, generally preferring small rivers and streams with constant flow through the year and certain slope to favor oxygenation of the waters (Charbonnel et al., 2015; Palmeirim & Hoffmann, 1983); this makes this species an ideal model to understand the interaction between genetic structure and dispersal patterns across rivers and land. The species presents strong adaptations to the aquatic ecosystems such as a highly mobile protracted snout, large hindfeet, and a long tail with stiff hairs (Palmeirim & Hoffmann, 1983). Due the fragility of its habitat, this species is endangered in part of its distribution range and is considered as Vulnerable at the global scale by the IUCN red list (Fernandes, Herrero, Aulagnier, & Amori, 2011). According to the IUCN, major threats include water pollution, increased water extraction for irrigation, and habitat fragmentation due to the construction of hydroelectric plants and dams. In addition, predation by American mink (*Neovison vison*) might be affecting populations where this invasive species is abundant (Romero, 2015). Climate change is also anticipated to be a threat to the desman in the future and, given climate scenarios for the Iberian Peninsula, the distribution of the species and its habitat may experience significant decreases in the most vulnerable areas (Fernandes et al., 2011; Morueta‐Holme, Fløjgaard, & Svenning, 2010; Thomas, Griffiths, & Ormerod, 2016). Previous studies on the genetic structure and diversity of this species revealed a strong phylogeographic structure (Igea et al., 2013; Querejeta et al., 2016). According to mitochondrial data, there are two main groups (A and B), which are further subdivided into four subgroups (A1, A2, B1, and B2) of allopatric distribution and likely glacial origin (Igea et al., 2013). The populations with the largest genetic diversity were detected in the occidental part of the distribution, thereby suggesting that one of the most important glacial refugia was in this area. Interestingly, the contact zones between the main mitochondrial groups (one located in the Cantabrian Mountains and the other in the Iberian Range) indicated an almost complete absence of spatial mixing between these maternal lineages after postglacial recolonization (Igea et al., 2013). A genomic analysis based on ddRAD sequences and SNPs corroborated the existence of a strong structure (Querejeta et al., 2016). The high genetic diversity of the occidental population compared to other populations was confirmed by the same genomic analysis (Querejeta et al., 2016).

Previous studies showed that the overall genetic structure of the Pyrenean desman was basically associated with the main mountain ranges, whereas the influence of river basins on the partition of the genetic diversity was smaller than previously believed (Igea et al., 2013; Querejeta et al., 2016). However, the sampling density in these studies was low and the study of the effects that rivers may have on the genetic structure at smaller scales was not possible. In this work, we obtained an extensive sampling of the Pyrenean desman in the occidental population thanks to different types of noninvasive samples and studied how the river networks shaped the phylogeographic pattern of this semiaquatic species. We aimed to answer two basic questions: (1) What is the genetic structure and the variability of the genetic diversity in the Pyrenean desman in the northwestern region of the Iberian Peninsula, and (2) whether this genetic structure is more influenced by overland or river dispersal.

**Figure 1:**
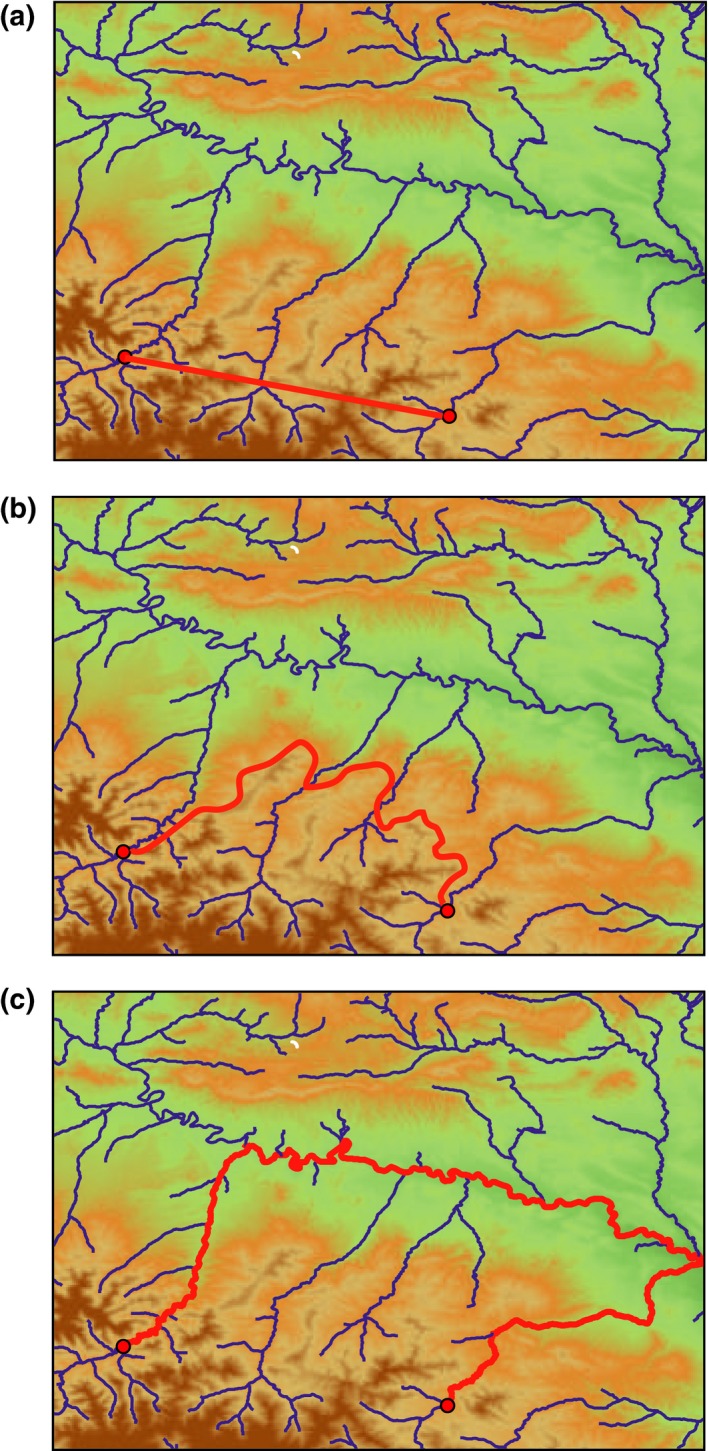
Scheme of the computation of Euclidean (a), least‐cost path (b), and river (c) distances between two sampling points

Our approach derives from the well‐known isolation‐by‐distance model (Wright, 1943), which is based on the premise that genetic isolation increases with geographical distance and predicts genetic similarity at shorter distances as well as differentiation at greater distances due to limited dispersal. In principle, Euclidean distances can be used to infer isolation by distance for terrestrial organisms. However, it was observed that distances that account for the landscape structure can better predict isolation by distance (Coulon et al., 2004). Furthermore, for aquatic and semiaquatic organisms, distances across the river network should be taken into account (Vignieri, 2005). Following these previous works, we studied for the occidental clade of the Pyrenean desman the correlation between genetic distance and three kinds of geographical distances. Firstly, we calculated the Euclidean distance, as a simple measure of the geographical distance between two samples. We then calculated a least‐cost path (Adriaensen et al., 2003) that accounts for altitude as only covariate, as a better approximation for the geographical distance in mountain areas. Finally, we calculated the river distance along the river networks, which we hypothesized to be a highly important variable for a semiaquatic species (Figure 1). A high correlation between genetic distance and Euclidean and/or altitudinal distance would be consistent with an important effect of isolation by distance and overall dispersal pattern taking place overland, whereas a high correlation between genetic distance and river distance would suggest isolation by distance with average dispersal occurring along rivers. We also discuss the interest of this approach to discern the evolutionary and ecological factors involved in the dispersal of semiaquatic species and the importance that this knowledge may have for conservation planning of the Pyrenean desman.

## 2 METHODS

### 2.1 Sampling, DNA extraction, and amplification

A total of 192 samples from Pyrenean desmans of the northwestern part of the Iberian Peninsula (Spain and Portugal) were used (see Table S1 and Figure S1 of Supporting Information). Most of these samples (131) were collected as part of two LIFE+ projects dedicated to the study of the Pyrenean desman: DESMANIA (LIFE11 NAT/ES/000691) and MARGALULLA (LIFE09 NAT/ES/000514). Of these samples, 84 were obtained from feces that desmans deposit on emerged rocks in the rivers. A further 47 samples were from depredated desmans found in the excrements of carnivores, likely Eurasian otters, which were collected in rivers (Callejo & Delibes, 1987). The dataset was complemented with the sequences from 61 samples of a previous study of the Pyrenean desman (Igea et al., 2013).

DNA of the new samples was extracted using the DNeasy Blood and Tissue kit from QIAGEN, following the manufacturer's instructions, in a separated UV‐irradiated area with dedicated equipment. Desman feces were treated as described in Igea et al. (2013). Carnivore feces containing depredated desmans were cleaned, and hair and tissue of desman origin were separated for extraction. This material was then processed as for the other samples.

The last 724 bp of the mitochondrial cytochrome *b* gene, which is the fragment with the highest number of variable positions, was amplified using two PCRs, and the first 342 bp of the D‐loop fragment was amplified in another PCR. PCR conditions, primers, and sequencing procedures can be found in Igea et al. (2013). The sequences were edited and assembled using genious pro (Biomatters Ltd). The final sequences of both genes obtained in this and previous work were concatenated, resulting in an alignment of 1,066 bp for 192 sequences. New sequences were deposited in the European Nucleotide Archive/GenBank under accession numbers LT799463–LT799724.

### 2.2 Phylogenetic analyses

The concatenated alignment of cytochrome *b* and the D‐loop was used to reconstruct a maximum‐likelihood phylogenetic tree using phyML 3.0 (Guindon et al., 2010). An HKY model was used following the work of Igea et al. (2013). A haplotype genealogy was obtained based on the reconstructed tree using haploviewer 1.0 (Salzburger, Ewing, & von Haeseler, 2011).

Our analysis confirmed the existence of two main mitochondrial groups in the area, A and B, with a mostly allopatric distribution. Subsequent analyses were performed with clade A, which consisted of 157 samples, to avoid the confounding effects of the more divergent sequences from clade B.

### 2.3 Genetic diversity

To explore the genetic diversity of the Pyrenean desman in the studied area, we used the alignment and the sample coordinates to compute, for each sampling point, the nucleotide diversity (π) values for all samples found in a radius of 10 km around the sampling point (Igea et al., 2013). We then used an inverse distance weight algorithm to interpolate the values of π in a grid of 1 × 1 km of the distribution studied, using the *idw* function of the R package gstat (Pebesma & Graeler, 2016). The surface obtained was superimposed on a map of the distribution studied. In addition, we obtained the unique haplotypes within the alignment using the *FindHaplo* function of the R package sidier (Muñoz‐Pajares, 2013) and also plotted them on the map.

### 2.4 Analysis of molecular variance

To study the extent to which river basins determine the genetic structure, we conducted a hierarchical analysis of molecular variance (AMOVA) (Excoffier, Smouse, & Quattro, 1992) using a matrix of pairwise genetic distances and the isolated river basins in which the samples were found as unique factor. This analysis was performed using the R package pegas (Paradis, 2010).

The matrix of genetic distances between all samples was calculated as the sum of the branch lengths separating them in the maximum‐likelihood tree (patristic or phylogenetic distances), which should account for all the divergence among sequences. We used the *cophenetic.phylo* function of the R package ape for this calculation (Paradis, 2004).

### 2.5 Geographical distances

Before computing the geographical distances, sample coordinates were moved to the nearest point in the river using the *snapPointsToLines* function of the R package maptools (Bivand et al., 2016).

Euclidean pairwise distances were calculated using the *pointDistance* function of the R package raster (Hijmans et al., 2016), which takes into account the curvature of the Earth.

The least‐cost path was computed using the R package gdistance (van Etten, 2015). We first calculated the transition matrix from a raster of altitudes taken from WorldClim (http://www.worldclim.org) using the *transition* function and then applied a correction to take into account the curvature of the Earth with the *geoCorrection* function. Finally, the least‐cost path was calculated using the *costDistance* function.

River distances were computed using the R package secrlinear (Efford, 2015). First, a shapefile of the river network from the distribution studied was obtained by merging the river shapefiles from Spain (Ministerio de Agricultura, Alimentación y Medio Ambiente) and Portugal (Atlas do Ambiente Digital‐Instituto do Ambiente). This shapefile was converted to an R object of *linearmask* type using the *read.linearmask* function. The pairwise distance matrix was then obtained using the *netwotkdistance* function, which computes the shortest paths along the network. When two points belonged to different basins, and therefore unconnected river networks, the pair was treated as missing data in the correlation analyses.

Correlations between phylogenetic distance and the three types of geographical distances were calculated by means of a Mantel test using the *mantel.test* function of the R package ncf (Bjornstad, 2016), for a total of 9,999 permutations.

## 3 RESULTS

### 3.1 Phylogenetic analysis

**Figure 2:**
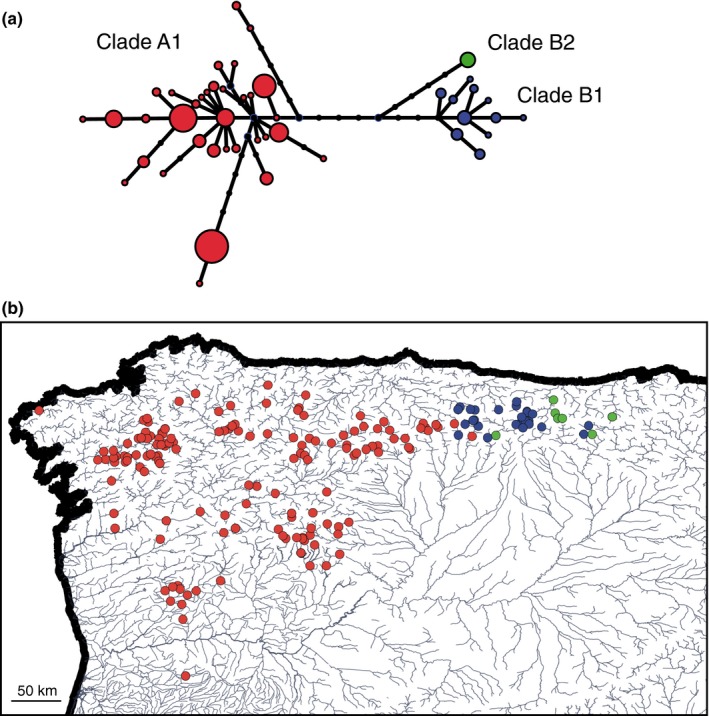
Haplotype genealogy of the mitochondrial sequences based on a maximum‐likelihood tree in which circles represent haplotypes, size is proportional to the number of individuals, and black dots represent intermediate, unsampled haplotypes (a), and map of the samples colored according to the mitochondrial clade (b)

The haplotype genealogy based on the maximum‐likelihood tree (Figure 2a) showed a clear separation between clades A (with samples belonging to subgroup A1) and B (with samples from subgroups B1 and B2). The visualization of the main clades in the map (Figure 2b) confirmed a strict geographical separation between them despite the large number of samples used. Thus, clade A1 occupies the occidental part of the studied area and clade B1 the northeastern part, with almost no mixing between them. Specimens from both clades were found in only two rivers (Curueño and Porma; Figure S2). A few samples from clade B2 are intermixed with B1 samples, as reported previously (Igea et al., 2013).

### 3.2 Genetic diversity

**Figure 3:**
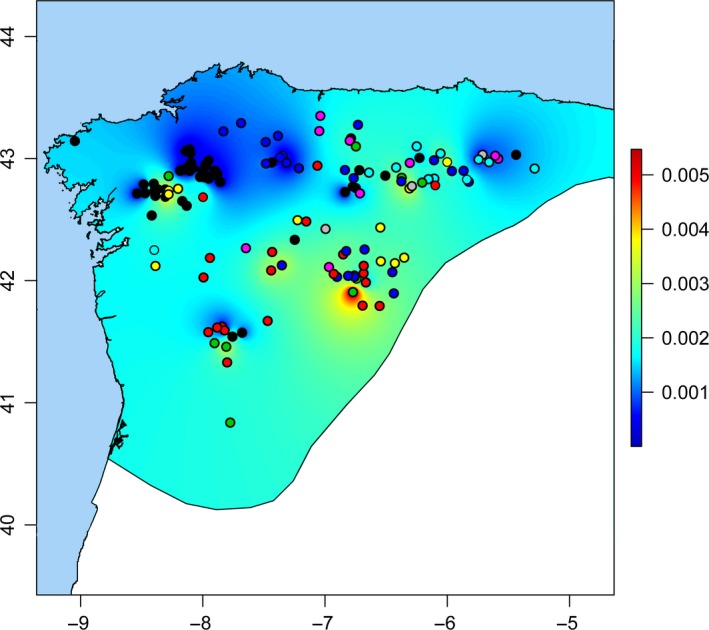
Plot of interpolated nucleotide diversity values, with the color scale indicating nucleotide diversity. Different haplotypes are shown with different colors. A different random color is used for each haplotype, and therefore, a greater variety of colors in an area also indicates greater genetic diversity

Genetic diversity was analyzed using 157 samples from clade A1. Both the plot of interpolated nucleotide diversity values and the distribution of different haplotypes (Figure 3) revealed that genetic diversity is not homogeneous across the area, with spots of low and high diversity being found in the distribution studied. When averages per basin were determined, nucleotide diversity showed the highest value in the Duero basin (0.00547). The lowest average was found in the Ulla basin (0.00019), where a large number of identical haplotypes were present.

### 3.3 Effect of river basin on genetic structure

The one‐way AMOVA performed using samples from clade A1 and the 11 isolated river basins as a factor (Table S1 and Figure S1) revealed that 53.8% of the genetic variation was due to the partition between river basins (Table S2; *p* > .05).

### 3.4 Dispersal patterns

We then performed the Mantel tests for all samples from clade A1 and found a correlation of 0.32 between the phylogenetic and Euclidean distances and 0.48 between the phylogenetic and least‐cost path distances, with both having significant *p*‐values (*p* < .05; Table 1). The correlation between phylogenetic and river distances was not significant.

**Table 1:** Results obtained from the Mantel tests for all the distance matrices. Altitude refers to the altitude‐based least‐cost path

Distances compared	Basin	Correlation	*p*‐Value
Euclidean vs. Phylogenetic	All	.32	.0002
Altitude vs. Phylogenetic	All	.48	.0002
River vs. Phylogenetic	All	−.06	.0533
Euclidean vs. Phylogenetic	Duero	.11	.0568
Altitude vs. Phylogenetic	Duero	.16	.0290
River vs. Phylogenetic	Duero	.02	.2578
Euclidean vs. Phylogenetic	Miño	.40	.0002
Altitude vs. Phylogenetic	Miño	.41	.0002
River vs. Phylogenetic	Miño	.38	.0002

We also performed the correlation analyses in the two basins with the largest number of samples. The correlation between the phylogenetic and Euclidean distances in the Duero basin (Figure S1), with 55 samples, was 0.11 and that between the phylogenetic and least‐cost path distances was 0.16, although only the latter was significant (Table 1). The correlation between the phylogenetic and river distances was not significant.

In contrast, the three Mantel tests for the Miño basin (Figure S1), with 44 samples, were all significant (Table 1), with a value of 0.40 for the correlation between phylogenetic and Euclidean distances, 0.41 when the least‐cost paths were considered, and 0.38 for river distances.

## 4 DISCUSSION

### 4.1 Phylogeographic structure and strict contact zone in the Cantabrian Mountains

The Pyrenean desman shows a strong genetic structure, probably due to the past existence of several isolated glacial refugia in which different populations persisted during Pleistocene glaciations and accumulated genetic divergence (Igea et al., 2013; Querejeta et al., 2016). The existence of multiple glacial refugia within South European peninsulas, classically considered to be homogeneous refugia, has been proven for many other endemic and semiendemic species (Alexandrino, Froufe, Arntzen, & Ferrand, 2000; Centeno‐Cuadros, Delibes, & Godoy, 2009; Godinho, Crespo, & Ferrand, 2008; Gomez & Lunt, 2007; Gonçalves et al., 2009; Martinez‐Solano, Teixeira, Buckley, & García‐París, 2006; Razgour, Salicini, Ibáñez, Randi, & Juste, 2015). This list includes species associated with aquatic ecosystems such as the golden‐striped salamander *Chioglossa lusitanica* (Alexandrino et al., 2000) or the southern water vole *Arvicola sapidus* (Centeno‐Cuadros et al., 2009). In this work, we used a large sample set of the Pyrenean desman from the occidental part of the Iberian Peninsula, which includes the two main mitochondrial clades of this species and the contact zone between them, to determine whether a larger dataset could help to reveal some spatial intermixing of clades and to study how dispersal across land and rivers may have shaped the genetic structure of this semiaquatic species. Specifically, the mitochondrial clades A1 and B1 likely entered into contact in the Cantabrian Mountains after the postglacial recolonization from their respective refugia (Igea et al., 2013). The detailed genetic characterization of these populations carried out in this work allowed us to detect the contact zone between these clades and revealed that there is indeed a small degree of spatial mixing between them (Figure S2). Specimens belonging to the two clades were only found in two rivers, namely the Porma and its tributary the Curueño. The fact that no other specimen of clade A1 can be found in the area of clade B1, and vice versa, supports a strong phylogeographic structure for this species (Igea et al., 2013). The distribution pattern of both mitochondrial clades is intriguing because there is no apparent physical barrier to dispersal in this contact zone. Future in‐depth analyses in this contact zone, including genomic analysis, may reveal crucial aspects concerning contemporary dispersal and gene flow, thereby helping to better delimit conservation units (Crandall, Bininda‐Emonds, Mace, & Wayne, 2000).

### 4.2 Variability of the genetic diversity and glacial history

The spatial analysis of the genetic diversity of clade A1 showed several areas of high genetic diversity (Figure 3). In particular, the area with highest values (>0.004) is located in the center of the studied area. The species‐distribution modeling performed in a previous work revealed that the areas of maximum probability of potential distribution during the last glacial maximum were largely coincident with areas of maximum genetic diversity (Igea et al., 2013). However, according to such study, the optimal glacial refugium of the northwestern area was closer to the coast than the high genetic diversity area observed here. The high mitochondrial diversity observed in these areas displaced from the original refugia could be partly explained by mixing of haplotypes coming from different regions. In any case, the study of such details about the glacial history of the Pyrenean desman will require a more extensive sampling in this area and, particularly, the use of genomic data to summarize genetic diversity from more loci.

Spots of low genetic diversity can be observed in the contact zone of clades A1 and B1 in the Cantabrian Mountains and in another area in the most northwestern part (Ulla basin) (Figure 3), which, in principle, may correspond to areas recently colonized via a bottleneck (Gomez & Lunt, 2007; Hewitt, 2000; Taberlet, Fumagalli, Wust‐Saucy, & Cosson, 1998). The contact zone between the mitochondrial clades may have been subjected to harsh conditions during the last glacial maximum (Serrano, González Trueba, Pellitero, & Gómez‐Lende, 2016) and therefore is likely to have been only recently colonized (Igea et al., 2013). However, the Ulla basin is close to areas of potential distribution during the last glacial maximum (Igea et al., 2013). Therefore, a more complex hypothesis that includes the existence of a glacial refugium, local extinctions and a subsequent recolonization via a bottleneck, may be necessary to explain the current low genetic diversity in this basin. These complex scenarios, also suggested for other species of the Iberian Peninsula, would give further support to the intricate evolution of populations associated with the glacial history of South European peninsulas (Godinho et al., 2008; Gomez & Lunt, 2007).

### 4.3 Relative importance of dispersal modes in semiaquatic species

The AMOVA revealed that only 53.8% of the genetic variability was due to the isolated river basins, thus indicating that, although these basins may have played an important role in structuring the genetic diversity, the desmans are not completely isolated within them.

The Mantel tests revealed a high correlation between overland distances (both Euclidean and least‐cost path) and phylogenetic distances for the whole area studied, thus indicating a strong effect of isolation by distance in the Pyrenean desman. The main implication of this result is that dispersal is low in this species. This conclusion is in agreement with data known from radiotracking studies, which showed that desmans have reduced vital areas usually including only hundreds of meters in a river stretch and limited dispersal capability (Melero, Aymerich, Luque‐Larena, & Gosálbez, 2012; Melero, Aymerich, Santulli, & Gosálbez, 2014; Stone, 1987). Moreover, a study based on genotyping of feces showed that long‐range movement was exceptional in this species (Gillet et al., 2016).

The correlation found with overland distances but not river distances in the whole area studied indicates that the isolation‐by‐distance pattern can be better modeled in this area, in principle, through terrestrial dispersal in the Pyrenean desman. In addition, the lack of correlation between river and phylogenetic distances suggests that the effects of dispersal along the river network were eclipsed by overland dispersal. These results, and particularly the lack of correlation between river and genetic distances, are in apparent contradiction with the known dependency of the Pyrenean desman on the aquatic medium. However, they may be explained by past climate conditions. Specifically, it is possible that desmans colonized new basins from the glacial refugia using terrestrial or riparian corridors during the Holocene (Igea et al., 2013). Overland dispersal may have been facilitated during this period due to deglaciation and the subdivision of large glaciers into smaller ones (Serrano et al., 2016), likely giving rise to a greater abundance of streams and humid habitats. The late Holocene has also been associated with a greater fluvial activity in the Iberian Peninsula (Thorndycraft & Benito, 2006). It is therefore possible that the river network was larger than it is today and that rivers reached higher parts of the mountains so that the distance between headwaters was shorter, thus facilitating connectivity between basins and sub‐basins. This way, overland dispersal of the Pyrenean desman, would have been reduced to short stretches between basins. Furthermore, these favorable conditions could have been maintained over a long period of the year due to increased precipitation (Thorndycraft & Benito, 2006). Therefore, the Holocene may have been highly favorable for the dispersal and colonization of large areas by a semiaquatic species like the Pyrenean desman. It is also interesting to note that connections between certain noroccidental basins have been proposed to exist during glacial periods in order to explain the distribution of some Iberian fishes (Aboim, Cunha, & Coelho, 2009; Aboim, Mesquita, Drago, Coelho, & Alves, 2013). Their effects on the overall dispersal patterns of the Pyrenean desman cannot be discarded although they may be difficult to proof for this semiaquatic species, which can use both terrestrial and river dispersal, in contrast with fishes, which can only disperse through the aquatic network. In any case, all these historical factors may help explain the lack of correlation observed between river and genetic distances.

Similar results were found for the Duero basin, which is the largest one in the area studied. A significant correlation was found between phylogenetic distances and least‐cost path distances but not river distances. The correlations were low in this basin, implying a relatively marginal isolation‐by‐distance pattern at this level. These weak correlations may be caused by the particular distribution of our samples in this basin, which only covers its northwestern edge and does not include most of the basin (Figure S1). In any case, these results are again consistent with the colonization of this large basin via an important component of overland dispersal, also likely to have taken place during the Holocene deglaciation period.

In contrast, a significant correlation between phylogenetic distances and both overland and river distances was found for the Miño basin, as expected for a semiaquatic mammal. The small mountains in this basin and the presence of corridors at low altitudes may have favored multiple overland interconnections between rivers, thus explaining the correlation between phylogenetic and overland distances. However, the presence of a significant correlation between phylogenetic and river distances indicates a prominent effect of river dispersal. The smaller size of this basin makes it likely that these correlations were less influenced by large postglacial colonizations, as compared to larger basins and the whole area studied, thus allowing to detect the overall effects of both river and overland dispersal.

To explain the different dispersal modes observed for the Pyrenean desman in the Duero and Miño basins, important aspects other than differences in basin size should be taken into account. Within the Duero basin, the Pyrenean desman is present mainly in the headwaters of rivers, where it can find suitable habitats, whereas large tributaries and rivers present in the central part of this basin are not adequate for the species, as indicated by its absence in this area (Figure S1). This central area in the Duero basin is likely to interrupt dispersal and break the correlation between phylogenetic and river distances and, probably, also contributes to the low correlation observed between phylogenetic and terrestrial distances. The Miño basin, however, has suitable riverine habitats for the desman in the majority of the basin, thus facilitating interconnection of desmans through a larger part of the river network and explaining the significant correlation between phylogenetic and river distances.

It is also important to consider that rivers inhabited by the Pyrenean desman are interrupted by artificial barriers that include hydroelectric plants and dams, but also ecological barriers caused by water pollution and desiccation due to water extractions (Fernandes et al., 2011). This is creating fragmented populations with reduced connectivity, which, in turn, may have affected the calculation of the correlations between phylogenetic and river distances; this is a problem that we could not avoid in this work although, being recent barriers, they could have had a small effect at the level of the relatively ancient genetic structure studied in this work. Specific studies aimed to investigate these water infrastructures will be necessary to understand how they affect recent dispersal of the Pyrenean desman.

Taken together, these results show a strong isolation‐by‐distance effect in the Pyrenean desman that can in principle be modeled through certain degree of overall overland dispersal when large areas are considered, and through both river and overland dispersal when smaller basins with highly appropriate habitats are analyzed; when a large proportion of the analyzed area is unsuitable for the species, the isolation‐by‐distance effect detected may be smaller. These results also suggest that the postglacial colonization of new areas was quite successful during the deglaciation phase, in which favorable habitats for the desman may have been highly abundant, thus leading to the rapid colonization of large areas that constitute the current species range. This dispersal may have occurred through overland corridors between basins and a more extensive fluvial network that may have been present during the Holocene (Thorndycraft & Benito, 2006). It is likely that contemporary inter‐river dispersal became more reduced after this period. This restricted dispersal would explain the lack of current spatial mixing of mitochondrial clades in their contact zone despite the absence of any clear geographical barrier (Figure S2).

The methodology used here to study these dispersal modes, and the results obtained, open up a promising avenue of research regarding the study of evolutionary and ecological factors that affect dispersal in different semiaquatic species. Thus, these methods can complement previous studies on semiaquatic species that also disperse along rivers or land depending on different ecological factors, such as the Eurasian otter (Pagacz, 2016), the Eurasian beaver (Senn et al., 2014), or the platypus (Furlan et al., 2013), to name a few, and determine to what extent they depend on river and overland dispersal modes. As we have shown here, it is important to note that the results of this isolation‐by‐distance method may be dependent on the size, geography, and ecological features of the area analyzed, and, consequently, different scales may reflect different aspects of the evolutionary history and ecology of semiaquatic species.

It should also be taken into account that the divergence levels in mitochondrial DNA analyzed herein arose during ancient periods, and therefore, the dispersal patterns inferred with these data apply mainly to them (Avise, 2009). Furthermore, it is important to consider that this isolation‐by‐distance method allows detecting overall dispersal patterns but not specific dispersal routes. The study of more specific contemporary dispersal patterns would require an analysis of a higher number of nuclear loci to reveal fine‐scale genetic structure. The computation of genetic distances derived from the nuclear genome may also help to avoid problems with dispersal differences between sexes because mitochondrial DNA is only inherited by females and would not allow different patterns of male dispersal to be determined (Nater et al., 2011; Waits, Taberlet, Swenson, Sandegren, & Franzén, 2000). However, both mitochondrial and genomic studies provide complementary data and are necessary to understand both the evolutionary history of the species and its dispersal behavior.

### 4.4 Conservation implications

Studies of dispersal patterns are especially relevant for endangered species like the Pyrenean desman. Without doubt, preserving and restoring the aquatic ecosystem is the most important aspect for the conservation of this semiaquatic species across its whole range. However, our results suggest that certain degree of interbasin dispersal was important in the recent evolution of the species in the northwestern region and therefore reinforce the idea that riparian and terrestrial corridors between isolated river basins should also be protected because they have a great potential for the dispersal of the species in this area. In particular, riverine habitats of the headwaters of rivers, especially those situated at low altitudes and with tributaries close to the watershed divides, may facilitate genetic interchange between populations and therefore deserve special protection. In addition, conservation of the habitats near watershed divides, including forests, shrub areas, peatlands, and even mountain pastures, may also be important to favor inter‐river dispersal. Ultimately, an effective protection of the whole riverine ecosystem that includes rivers, upland streams, and potential corridors between them may be essential for the long‐term survival of the Pyrenean desman.

Finally, a note of caution is needed because this study was carried out in an area that mostly includes relatively small mountains with multiple connections between them. However, the Pyrenean desman is also present in rivers and streams of higher mountain ranges. Hence, additional studies are required in other populations of the species such as those near the high mountains of the Pyrenees, the Cantabrian Mountains, or the Central System, where connections between basins and sub‐basins may be more rare or extremely difficult to cross for this species. In these areas, dispersal patterns, connectivity, and the genetic structure of the Pyrenean desman could be different to the ones observed in this work, and, consequently, it is possible that other important conservation problems of the species are revealed in them. In summary, more detailed studies are needed to better understand the overall species requirements and design the best possible conservation strategies.

## CONFLICT OF INTEREST

None declared.

## AUTHOR CONTRIBUTIONS

J.C. and M.Q. conceived the ideas; A.F.‐G. and R.R. collected the samples; M.Q. performed the laboratory work and analyzed the data; and M.Q. and J.C. led the writing with input from the other authors.
