# Supplementary material for: Postglacial dispersal patterns and mitochondrial genetic structure of the Pyrenean desman (Galemys pyrenaicus) in the northwestern region of the Iberian Peninsula
Source: Ecol Evol. 2017 May 15;7(12):4486–95. doi: 10.1002/ece3.3034 (PMC5478051; doi:10.1002/ece3.3034)
Supplement: Supplementary file 1 [file ECE3-7-4486-s001.pdf]

SUPPORTING INFORMATION

Ecology and Evolution

Postglacial dispersal patterns and mitochondrial genetic structure of the Pyrenean desman (*Galemys pyrenaicus*) in the north-western region of the Iberian Peninsula

Marina Querejeta, Angel Fernández-González, Rafael Romero and Jose Castresana

Table S1. Samples used in this study, including those also used in a previous study.

Specimen code	Latitude	Longitude	Basin	Sample type
IBE-BC0064	42.69	-8.31	Ulla	Otter faeces
IBE-BC0093	42.01	-6.74	Duero	Desman faeces
IBE-BC0123	42.48	-7.15	Miño	Otter faeces
IBE-BC0129	42.01	-6.74	Duero	Desman faeces
IBE-BC0204	42.26	-6.68	Miño	Desman faeces
IBE-BC0256	43.00	-5.69	Duero	Desman faeces
IBE-BC0259	42.92	-5.29	Duero	Desman faeces
IBE-BC0269	42.22	-6.85	Miño	Desman faeces
IBE-BC0311	42.06	-6.69	Duero	Desman faeces
IBE-BC0370	42.04	-6.76	Duero	Desman faeces
IBE-BC0377	43.02	-5.61	Duero	Desman faeces
IBE-BC0384	42.11	-6.97	Miño	Desman faeces
IBE-BC0388	42.03	-6.90	Duero	Desman faeces
IBE-BC0389	42.06	-6.93	Duero	Desman faeces
IBE-BC0401	42.03	-6.76	Duero	Desman faeces
IBE-BC0472	42.90	-5.87	Duero	Desman faeces
IBE-BC0476	41.90	-6.44	Duero	Desman faeces
IBE-BC0483	42.43	-6.55	Miño	Desman faeces
IBE-BC0497	42.84	-6.11	Duero	Desman faeces
IBE-BC0505	42.12	-6.68	Duero	Desman faeces
IBE-BC0508	42.14	-6.43	Duero	Desman faeces
IBE-BC0541	41.99	-6.67	Duero	Desman faeces
IBE-BC0542	42.16	-6.54	Duero	Desman faeces
IBE-BC0567	42.19	-6.35	Duero	Desman faeces
IBE-BC0586	42.93	-6.42	Miño	Desman faeces
IBE-BC0605	42.07	-6.45	Duero	Desman faeces
IBE-BC0615	43.03	-5.71	Duero	Desman faeces
IBE-BC0618	43.00	-5.58	Duero	Desman faeces
IBE-BC0623	42.90	-5.96	Duero	Desman faeces
IBE-BC0648	43.05	-4.85	Duero	Desman faeces

IBE-BC0652	43.05	-4.77	Duero	Desman faeces
IBE-BC0653	43.03	-5.44	Duero	Desman faeces
IBE-BC0692	43.16	-5.04	Sella	Desman faeces
IBE-BC0702	42.97	-5.66	Duero	Desman faeces
IBE-BC0718	43.02	-5.61	Duero	Desman faeces
IBE-BC0723	43.00	-6.23	Miño	Desman faeces
IBE-BC0725	42.99	-5.74	Duero	Desman faeces
IBE-BC0734	43.05	-4.82	Duero	Desman faeces
IBE-BC0780	43.03	-4.77	Duero	Desman faeces
IBE-BC0800	43.02	-5.35	Duero	Desman faeces
IBE-BC0803	42.83	-6.15	Duero	Desman faeces
IBE-BC0807	42.78	-6.10	Duero	Desman faeces
IBE-BC0809	42.80	-6.21	Duero	Desman faeces
IBE-BC0816	42.81	-5.82	Duero	Desman faeces
IBE-BC0830	42.84	-6.37	Miño	Desman faeces
IBE-BC0839	42.91	-6.84	Navia	Desman faeces
IBE-BC0843	42.84	-6.77	Miño	Desman faeces
IBE-BC0854	42.94	-4.24	Duero	Desman faeces
IBE-BC0858	42.90	-6.72	Miño	Desman faeces
IBE-BC0859	42.77	-6.78	Miño	Desman faeces
IBE-BC0869	42.89	-6.64	Miño	Desman faeces
IBE-BC0871	42.73	-6.84	Miño	Desman faeces
IBE-BC0887	43.00	-4.69	Duero	Desman faeces
IBE-BC0891	42.96	-6.31	Miño	Desman faeces
IBE-BC0893	42.83	-5.84	Duero	Desman faeces
IBE-BC0900	42.76	-6.31	Miño	Desman faeces
IBE-BC0945	42.92	-8.13	Ulla	Desman faeces
IBE-BC0948	43.04	-6.06	Duero	Desman faeces
IBE-BC0951	42.94	-4.32	Duero	Desman faeces
IBE-BC0956	42.99	-6.11	Duero	Desman faeces
IBE-BC0959	42.76	-6.75	Miño	Desman faeces
IBE-BC0961	42.77	-6.29	Miño	Desman faeces
IBE-BC0980	42.71	-6.71	Miño	Desman faeces

IBE-BC1008	42.86	-6.51	Miño	Desman faeces
IBE-BC1055	42.91	-5.15	Duero	Desman faeces
IBE-BC1077	42.93	-5.08	Duero	Desman faeces
IBE-C1154	43.17	-6.79	Navia	Igea et al. 2013
IBE-C1174	43.15	-6.80	Navia	Igea et al. 2013
IBE-C1177	43.15	-6.80	Navia	Igea et al. 2013
IBE-C1729	43.01	-4.27	Ebro	Igea et al. 2013
IBE-C1843	43.10	-6.75	Nalon	Igea et al. 2013
IBE-C1851	43.14	-4.81	Deva	Igea et al. 2013
IBE-C1869	43.28	-6.73	Navia	Igea et al. 2013
IBE-C2517	43.15	-5.41	Nalón	Igea et al. 2013
IBE-C2607	43.15	-5.26	Nalón	Igea et al. 2013
IBE-C2736	42.25	-8.40	Miño	Igea et al. 2013
IBE-C2737	42.75	-8.37	Ulla	Igea et al. 2013
IBE-C2738	42.75	-8.37	Ulla	Igea et al. 2013
IBE-C2739	42.08	-7.44	Duero	Igea et al. 2013
IBE-C2740	42.24	-6.83	Miño	Igea et al. 2013
IBE-C2741	42.24	-6.83	Miño	Igea et al. 2013
IBE-C2742	42.19	-7.94	Miño	Igea et al. 2013
IBE-C2743	42.19	-7.94	Miño	Igea et al. 2013
IBE-C2745	43.23	-7.05	Navia	Igea et al. 2013
IBE-C2747	42.24	-7.43	Miño	Igea et al. 2013
IBE-C2750	42.12	-8.39	Miño	Igea et al. 2013
IBE-C2751	42.13	-7.35	Miño	Igea et al. 2013
IBE-C2752	42.13	-7.35	Miño	Igea et al. 2013
IBE-C2753	42.94	-7.06	Navia	Igea et al. 2013
IBE-C2754	42.42	-7.00	Miño	Igea et al. 2013
IBE-C2856	43.12	-4.56	Deva	Igea et al. 2013
IBE-C2893	43.18	-4.90	Deva	Igea et al. 2013
IBE-C2894	43.22	-4.90	Deva	Igea et al. 2013
IBE-C2950	43.17	-5.39	Nalón	Igea et al. 2013
IBE-C2975	41.57	-7.68	Duero	Igea et al. 2013
IBE-C2990	41.62	-7.84	Duero	Igea et al. 2013

IBE-C3301	41.79	-6.55	Duero	Igea et al. 2013
IBE-C3303	40.84	-7.78	Duero	Igea et al. 2013
IBE-C3305	40.84	-7.78	Duero	Igea et al. 2013
IBE-C3338	43.08	-4.54	Deva	Igea et al. 2013
IBE-C3342	43.07	-4.50	Deva	Igea et al. 2013
IBE-C3522	41.91	-6.77	Duero	Igea et al. 2013
IBE-C3541	41.80	-6.69	Duero	Igea et al. 2013
IBE-C3542	42.92	-8.08	Ulla	Desman faeces
IBE-C3545	41.91	-6.77	Duero	Igea et al. 2013
IBE-C3557	43.35	-7.04	Eo	Desman faeces
IBE-C3568	42.04	-6.81	Duero	Desman faeces
IBE-C3602	41.33	-7.80	Duero	Igea et al. 2013
IBE-C3620	42.93	-5.40	Duero	Igea et al. 2013
IBE-C3623	43.15	-5.41	Nalón	Igea et al. 2013
IBE-C3638	41.91	-6.77	Duero	Igea et al. 2013
IBE-C3641	43.15	-5.30	Nalón	Igea et al. 2013
IBE-C3647	41.91	-6.77	Duero	Igea et al. 2013
IBE-C3795	42.69	-8.39	Ulla	Desman faeces
IBE-C3812	42.72	-8.47	Ulla	Desman faeces
IBE-C3813	42.87	-7.88	Ulla	Desman faeces
IBE-C3822	42.73	-8.40	Ulla	Desman faeces
IBE-C3828	42.75	-8.01	Ulla	Desman faeces
IBE-C3833	42.97	-8.10	Ulla	Desman faeces
IBE-C3834	42.90	-8.11	Ulla	Desman faeces
IBE-C3843	42.86	-8.28	Ulla	Desman faeces
IBE-C3848	42.79	-8.40	Ulla	Desman faeces
IBE-C3849	42.75	-8.20	Ulla	Desman faeces
IBE-C3852	42.74	-8.03	Ulla	Desman faeces
IBE-C3868	42.75	-8.11	Ulla	Desman faeces
IBE-C438	42.97	-6.00	Duero	Igea et al. 2013
IBE-C4402	43.22	-7.83	Miño	Otter faeces
IBE-C4403	42.53	-8.42	Lerez	Otter faeces
IBE-C4404	42.33	-7.25	Miño	Otter faeces

IBE-C4405	42.92	-7.21	Miño	Otter faeces
IBE-C4407	42.03	-7.99	Limia	Otter faeces
IBE-C4409	42.27	-7.65	Miño	Otter faeces
IBE-C4410	42.83	-7.95	Ulla	Otter faeces
IBE-C4411	43.14	-9.05	Grande	Otter faeces
IBE-C4412	42.50	-7.22	Miño	Otter faeces
IBE-C4413	43.04	-8.10	Tambre	Otter faeces
IBE-C4414	43.06	-8.14	Tambre	Otter faeces
IBE-C4415	43.07	-8.11	Tambre	Otter faeces
IBE-C4417	42.85	-8.00	Ulla	Otter faeces
IBE-C4418	43.13	-7.49	Miño	Otter faeces
IBE-C4420	43.19	-7.39	Miño	Otter faeces
IBE-C4422	42.99	-7.95	Ulla	Otter faeces
IBE-C4423	42.71	-8.54	Ulla	Otter faeces
IBE-C4424	42.90	-8.19	Ulla	Otter faeces
IBE-C4428	42.75	-8.31	Ulla	Otter faeces
IBE-C4429	42.98	-7.40	Miño	Otter faeces
IBE-C4431	42.97	-7.32	Miño	Otter faeces
IBE-C4433	42.97	-7.44	Miño	Otter faeces
IBE-C4435	43.29	-7.69	Miño	Otter faeces
IBE-C4437	43.02	-7.35	Miño	Otter faeces
IBE-C4438	43.01	-7.37	Miño	Otter faeces
IBE-C4440	42.96	-7.48	Miño	Otter faeces
IBE-C4441	42.81	-7.86	Ulla	Otter faeces
IBE-C4442	42.86	-7.96	Ulla	Otter faeces
IBE-C4449	42.95	-7.98	Ulla	Otter faeces
IBE-C4451	42.76	-8.08	Ulla	Otter faeces
IBE-C4452	42.70	-8.01	Ulla	Otter faeces
IBE-C4453	42.68	-8.00	Ulla	Otter faeces
IBE-C4454	42.74	-8.28	Ulla	Otter faeces
IBE-C4459	42.65	-8.17	Ulla	Otter faeces
IBE-C4460	42.61	-8.13	Ulla	Otter faeces
IBE-C4461	42.94	-7.97	Ulla	Otter faeces

IBE-C4462	42.90	-8.01	Ulla	Otter faeces
IBE-C4465	42.98	-7.95	Ulla	Otter faeces
IBE-C4466	42.91	-8.16	Ulla	Otter faeces
IBE-C4470	42.79	-8.48	Ulla	Otter faeces
IBE-C4473	42.71	-8.28	Ulla	Otter faeces
IBE-C4474	42.85	-8.02	Ulla	Otter faeces
IBE-C4476	42.85	-7.99	Ulla	Otter faeces
IBE-C4477	42.86	-7.92	Ulla	Otter faeces
IBE-C4478	42.90	-7.90	Ulla	Otter faeces
IBE-C448	42.81	-6.38	Miño	Igea et al. 2013
IBE-C449	43.10	-6.25	Nalon	Igea et al. 2013
IBE-C539	43.24	-4.58	Deva	Igea et al. 2013
IBE-C618	43.09	-4.07	Duero	Igea et al. 2013
IBE-C683	43.09	-4.07	Saja	Igea et al. 2013
IBE-C720	43.13	-4.81	Deva	Igea et al. 2013
IBE-C723	43.11	-4.76	Deva	Igea et al. 2013
IBE-C779	41.58	-7.96	Duero	Igea et al. 2013
IBE-C802	41.59	-7.82	Duero	Igea et al. 2013
IBE-C831	41.62	-7.88	Duero	Igea et al. 2013
IBE-C844	41.67	-7.47	Duero	Igea et al. 2013
IBE-C894	41.54	-7.76	Duero	Igea et al. 2013
IBE-C895	41.46	-7.81	Duero	Igea et al. 2013
IBE-C919	41.54	-7.76	Duero	Igea et al. 2013
IBE-C922	41.49	-7.90	Duero	Igea et al. 2013
IBE-C975	43.09	-4.86	Duero	Igea et al. 2013
IBE-LE0402	43.01	-5.23	Duero	Desman faeces
IBE-LE0406	43.07	-5.27	Duero	Desman faeces
IBE-LE0411	43.06	-5.29	Duero	Desman faeces
IBE-LE0529	43.02	-4.90	Duero	Desman faeces
IBE-LE0536	42.99	-4.84	Duero	Desman faeces

Igea, J., P. Aymerich, A. Fernández-González, J. González-Esteban, A. Gómez, R. Alonso, J. Gosálbez, and J. Castresana. 2013. Phylogeography and postglacial expansion of the endangered semi-aquatic mammal *Galemys pyrenaicus*. BMC Evol. Biol. 13:115.

Table S2. Analysis of Molecular Variance (AMOVA) for the phylogenetic distances between desmans inhabiting isolated river basins and within desmans inhabiting isolated river basins. % is the percentage of variance explained by the isolation of river basins.

Source of variation	d.f.	SS	MS	Est. Variance	%	P-value
Among basins	9	0.0012429	0.0001381	0.0000102	53.8	***
Within basins	147	0.0012894	0.0000087	0.0000087	46.2	***
Total	156	0.0025324	0.0000162	0.0000189		

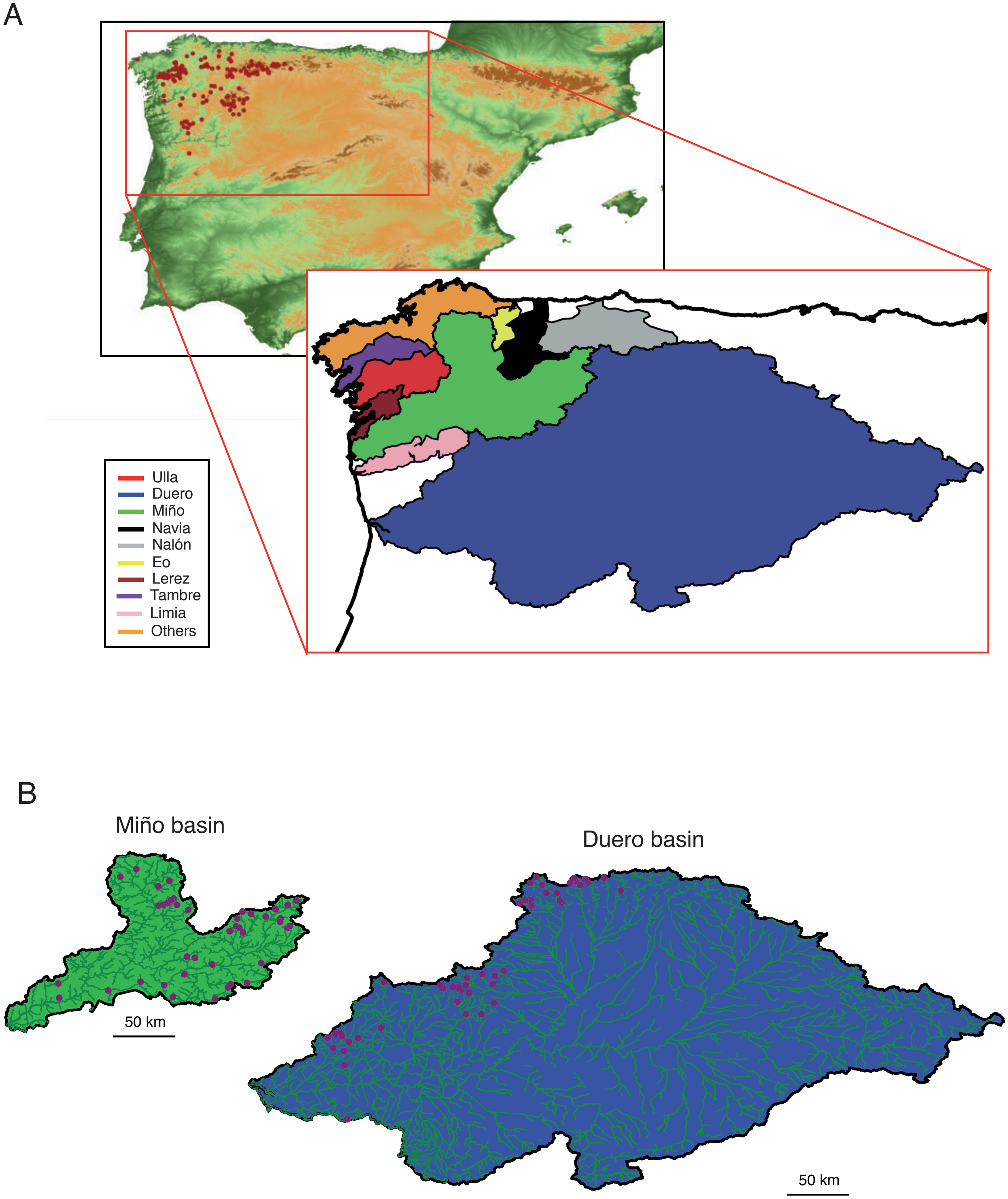

Figure S1. Map of the study area showing the main basins (A), and enlargement of the Miño and Duero basins (B). A number of smaller basins were grouped as “Others”.

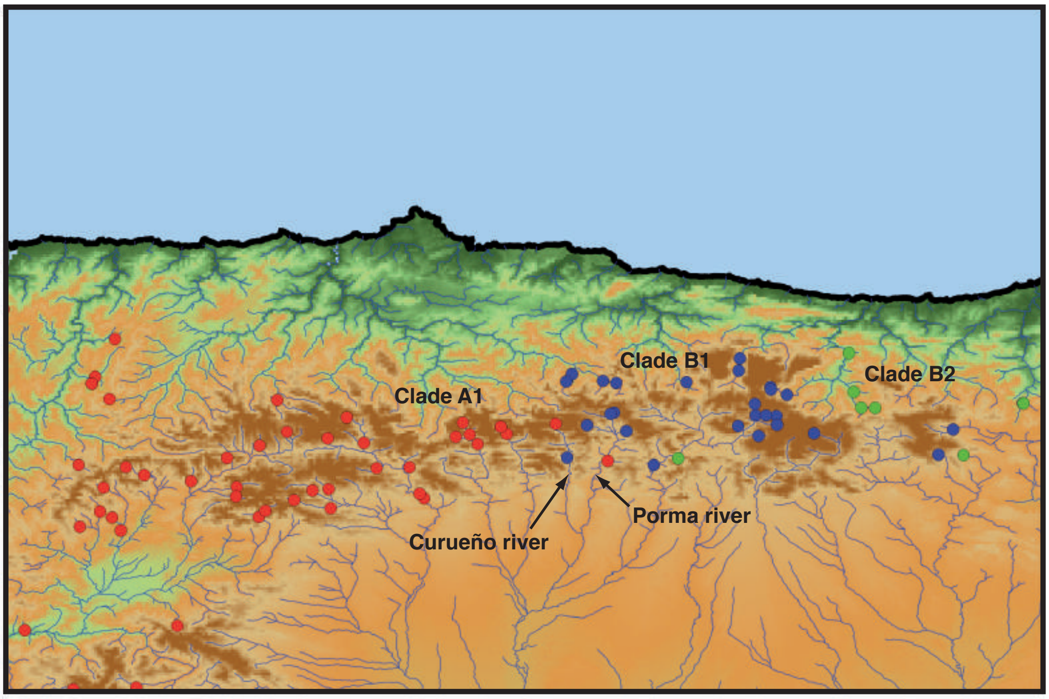

Figure S2. Enlargement of the contact zone between the main mitochondrial clades in the Cantabrian Mountains.
